# Table of Meteorological Conditions in the City of Buffalo for February, 1855

**Published:** 1855-04

**Authors:** Sanford B. Hunt


					﻿ART. X. —Record of Meteorological Conditions at Buffalo, for February,
1855. By Sanford B. Hunt, M. D.
2	s	s .
ca	“	go	2
ego
®	~	o>_-	Ph
a	o
«	£	®	m
<	02	>, ~ •g
s	c3	2 rZ	rt
a	•	8	bD
X	T 2 * °®	.2
a s	ts	t*
gpn	g	g
.__________________co o__________________tn________________________________________________________________________________cn_____
•ajnjv.iadcuaj, I
jo oStrej jsajBajgH1 «o n# <?j rH Mini' soon	t'tcHooowmtco h
•a’ii I ci	'C	ct	*-o	in >a o lc c'x o m o cs c in o 6o io >fl 6 tb io io	S
__________*• Jto	co	co	co_nn^n^o^n-imionr-i-iHmn^inooa) .__________at
-pnnnn mzaft; ~	-	o,	m	a	ocao<oooocagigig)<ocao>o>oa<ooaos <00000	o
>1	___„ Ct>	to	tO	irt	CO to 00 00 Cl CO Cl tO lO CO to «inCEOmCTt't"H<	ct
h?	?OI0 J	0 co to m . , totoooooc?c*5CMtomtoto . Cl IO 00 to CO Cl t— f~ f	o
5 -Ai9Q uBdu m> ci 00 co’ 00 tb —: uo ci ci i.c -c o mi 10 £- to —c ci tE ci x- t— o —1 —i	<—
g	*	, hH	t	hHh-.h-<—■dCOCOCOClCldClClClCl'-l________________________________________________________________.HrHrt_____.-t
|CO coco	co coco coco	to	co to	CO CO CO CO CO	.05
amps co	, . co co	co.cocococo	co	co	. co	, _ co co co co co	c-
-J9dtaaj,uBdjv|o 06	>0 co irf 00 oit^ciciibtbt^ciodoocnaoxPiboiboiocbm’irf	05
_____________C l hH —<____»	r- -, ^ O' 71 CO « « 7101 Cl Cl Cl CT Ct rH	______I r-|
| O '-O	O L.O	U0 H5 >.0 UO 1.0 C i-'. it C it it i'. O <O 10 10 uO o o~b CO	pO
-omrmnn-"	O CO	COCOCOCOCOCOCOCOCOCOCOCOCOCOCOCOCOCOOCOCO	Cl
u	Anpuxinj-l — —00	<0 0 O. O O X O C. X O O O CC C. C. ~. C. 'X O CO X	co
O________’—<__<	___ _____________________________ >—1	______I
c	CD	cd	0C£)^)C£)OCOCOCDCCCDCD^COC£>Q^(DCO CO CO	CQ
c .1UIO T M irT .	. CO *. tDCD<X>CDvDCQCDCDCO<DCDCDCOCDCDCDCDCQ . CQ 00	—<
c ♦ . a. U goh ci cd id ci id r*’ r- lq ci cd id r-’ r- oi rd r-’ co go
•	t£ ______—< r-<_____* ,-1	r-<	< GQ CO CO 04 04 04 04 04 GQ GQ 04_rH_________r-<
S £	i	CO
•	'Sim dmo t * •	•	• *. * •	•	••••••••	....•••.••.
C-i	‘	.GO O	C3 —1 r-f	GOiOOOOOOTf^GOGOOOHOOOO^^COCO	03
_________  J — 04______—<	—< 04 00	00 CO 04 04 00 00 04 CO Q4 ri r-i ri	j-i___
°	®	. pq W	.	.	....................................
§ SSgCjS	fe>tn«2^<n^^
.......... .. ............................................................ . ...................................
H _____________C -< it CT Cl	—<CIO-^—<—<—<Cl-f<CO—iOOOClrH.-<COin-H—<___________________________________________________________________
g spno|0jo;,uiv|ooo«oo 2~O22222O222OO2OO22°O	j
pj	o ut to lo ut io lo lo lo o to o o in to in <n c to to o o m m m it c c	PS
H	.  COCOCOCOCOCOCOCOCOCOCOCOi—icocococoococotocococococotoco	—1
e-.	. AJIPItnnH a> cs c; ci ci c: ci ct c> 00 c co co c~. c m ~ 0 0 c> co x ci a; ct a> as co	co
S  ----——------------------------------------------------------------------------------—_______________________________________
<->	-g	cocotocococotototocococo CO CO CO CO	tCCOCOCOCOCOCOCOCOCO	H
E ctuioj nan n co cc co to co to to ca com n , to co to to tocomcototocotococo	o
5 ■> ■ a. 11 -r o 00 cc cn t- c- co l-c t-2 -* -t< —r-l (< o co S ct c-^ ao — c-i c-’ <-	10
.<-.	r-l —I -H -I * _ _, „> r- CTCT CICtrlCTCTrtCT CTCTCT -•	!d
2 I? 7	*-----------------------------------------------------------------------------------------------------------«
. amj.duiax (jjotPco-HrPtocoe>c6o6c^-PtP'^ooo-Pocj'^o-4'^cocici	co
— ___________04 04 r-< —«	»—< 04 04 G^4 04 00 "rft CO CO CO CO CO CO CO 00 CO 04	04 04______04
o w 73	W	.......................................................................................
r=§§°2
..............................................................
______________________________________________________________tn 1Q IQ CO ^H(^M^r-IHHHH'^r-<COCOrH O GQ OJCO UO CO O?J-^____________
spno[Qjo44cav|o 2200<'dcoo2S222SS2222Sccc°(22S0223
lo m lo o 1.0	in l-o tn in vn o i.n id uo »-o id	in un	10	io »o	»o	uo m	»n	i>-
•XlTDTTim r j	O CO	CO CO CO CO CO tD COCO CO CO CO	CO CO	CO	CO CO	CO	CO CO	CO	CO
A4JPJUALHj	Q C7.	Q	Gj	GO	Gi Cl Q C O	Cl	Q	Q Gj	a	Q G.	Q	Ci
————CD CO CD	CO CO ‘A 'C O	'-C CD 'C O O C C C rC CO	QQ
g	D CD CD CD *	* COCOCOCDCDCOCOCDCOCOCOCOCDCOCOCDCDCOCOCD	rH
•	c ?uI°d M°Q 30 r-5 o co cd ei ci	cd co »d co’ 0 id cd -t	ci 1^- oi id oi ci qo
t£	—I — —.	—	-H^CO CO C? 04 GQ Q? G?
‘	‘	T
*G MIU,dure>L ,4 rji cri to to ci C5 t-'to'cj^oo^cocotojP^^tmoinooinocjzi
_______-71-- l—i	I—I -H H r~. Ct H1 m CT CT CT CT CT rH ft HH______________________________________________’—1 1—1 -H_rH_
5	<0 S -4	fe	...................................................fp^.................................
ogg=-S	02 co ai !?i	cocdfe^fS^pPpP
°	eo co ci co «■ U ci	-I h —1 h h-< -ici co coj-* O o hh ci ci co ci-1_________________________________________________
spnoiojoj.tav o to cn to ao co o g ° g g g S 2 2 ° 5 S ° 2 00	° S 2 S	|
T	------- ------—	jt©
00
t;	-utchjo-ui	ko’
h-j___________________________________________________________________________________________________________________________________! 
o	1 ao 00	ao	cn> 00 ci	ci	,	<05	.	.	.	ci c^ >-<	co co	.’“I .
*IH0 J®	1 rd rd	cd	rd GO	G"!	00	Oi	O"2	Oi	Cl Oi	O* 03	03 03 03
IgQc8g3	a?	rcgogjg	_G4G4G4GJ	Gt G4	0404_
•awrr(NW^LOCOt"00g>5z;£2322S222Sci&cicSciCT£g____________________________________________________________________________
* Below 0°.
				

